# Phylogeny-Directed Search for Murine Leukemia Virus-Like Retroviruses in Vertebrate Genomes and in Patients Suffering from Myalgic Encephalomyelitis/Chronic Fatigue Syndrome and Prostate Cancer

**DOI:** 10.1155/2011/341294

**Published:** 2011-09-04

**Authors:** Jonas Blomberg, Ali Sheikholvaezin, Amal Elfaitouri, Fredrik Blomberg, Anna Sjösten, Johan Mattson Ulfstedt, Rüdiger Pipkorn, Clas Källander, Christina Öhrmalm, Göran Sperber

**Affiliations:** ^1^Section of Clinical Microbiology, Department of Medical Sciences, Uppsala University, 751 05 Uppsala, Sweden; ^2^Clinical Microbiology, Academic Hospital, 751 85 Uppsala, Sweden; ^3^Deutsches Krebsforschungszentrum, Im Neuenheimer Feld 280, 69120 Heidelberg, Germany; ^4^Cavidi Tech AB, Uppsala Science Park, 751 83 Uppsala, Sweden; ^5^Department of Neuroscience, Biomedical Centre, Uppsala University, P.O. Box 593, 751 24 Uppsala, Sweden

## Abstract

Gammaretrovirus-like sequences occur in most vertebrate genomes. Murine Leukemia Virus (MLV) like retroviruses (MLLVs) are a subset, which may be pathogenic and spread cross-species. Retroviruses highly similar to MLLVs (xenotropic murine retrovirus related virus (XMRV) and Human Mouse retrovirus-like RetroViruses (HMRVs)) reported from patients suffering from prostate cancer (PC) and myalgic encephalomyelitis/chronic fatigue syndrome (ME/CFS) raise the possibility that also humans have been infected. Structurally intact, potentially infectious MLLVs occur in the genomes of some mammals, especially mouse. Mouse MLLVs contain three major groups. One, MERV G3, contained MLVs and XMRV/HMRV. Its presence in mouse DNA, and the abundance of xenotropic MLVs in biologicals, is a source of false positivity. Theoretically, XMRV/HMRV could be one of several MLLV transspecies infections. MLLV pathobiology and diversity indicate optimal strategies for investigating XMRV/HMRV in humans and raise ethical concerns. The alternatives that XMRV/HMRV may give a hard-to-detect “stealth” infection, or that XMRV/HMRV never reached humans, have to be considered.

## 1. Introduction

Recent reports of human gammaretroviruses highly similar to murine gammaretroviruses in PC and ME/CFS patients raise questions regarding (i) the occurrence of such retroviral sequences in murine and other vertebrate genomes, (ii) probable routes of spread of such viruses, and (iii) available methods for the detection of infection with them. In this review, we apply a phylogenetic aspect to the occurrence of XMRV/HMRV in genomes, and to the diagnostic search for it in humans. The comparative approach [1] can also enhance the study of pathobiology and epidemiology of XMRV/HMRV. Given the recent great activity in the field, the review cannot be exhaustive. Indeed, reports indicating that all XMRV/HMRV findings in humans may be due to different forms of laboratory contamination [2, 3] stress the need for a critical evaluation.

## 2. The Genus Gammaretrovirus

### 2.1. General

**Figure 1:**
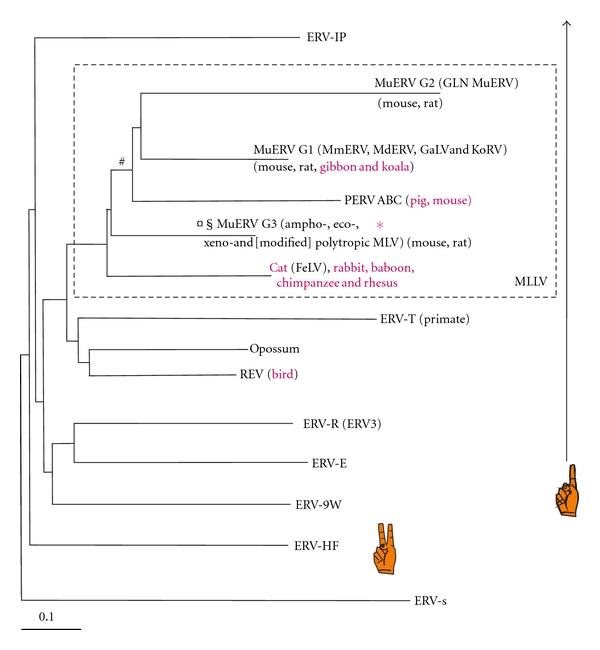
Simplified phylogeny of gammaretroviruses based on over 2000 gammaretrovirus-like sequences with one zinc finger in Gag, from selected vertebrate genomes (in a first version of RetroBank) and reference gammaretroviruses. Retroviral groups which occur in phylogenetically distant vertebrate hosts, indicating cross-species transmission events, are shown in red. The neighbor-joining tree was based on an alignment of 105 reference and consensus Pol amino acid sequences. Only the major branches are shown. As explained in the text, they were provisionally named after the cosegregating HERV group. The group of “MLV-like viruses,” MLLVs, at least 60% similar in Pol, is boxed in. The similarity is calculated from the inverted ratio of the BLASTP score of the sequence against itself to another sequence, or to a consensus Pol sequence. Some two-zinc finger sequences (ERV-HF, containing HERV-H and HERV-F) were included as a reference. Although the clustering included several genomes, the groups were named from the human sequence representative. MuERV: mouse endogenous retrovirus. MmERV is from Bromham et al. [13], and MdERV is *Mus dunni* ERV from Wolgamot et al. [14]. GLN MuERV is from Ribet et al. [15]. Symbols: ∗ position of XMRV/HMRV, § recombinant MuERV Sp496-5Sb [16] from *Mus spretus, ¤ hortulanus* endogenous murine virus, HEMV, from *Mus spicilegus* [17], # MuRRS [18], and MuERVC [19]. An ERV-S sequence [20] was used to root the tree. It came from HERV-S, a sequence intermediate to spuma-, epsilon-, and gammaretroviruses. It belongs to the so-called “ERV3 family” according to the RepBase nomenclature [21]. This large clade is also called “Class III ERVs.” It should not be confused with the ERV3 group shown in the figure. The hand symbols denote proviruses with one and two zinc fingers in Gag, respectively. The murine gammaretroviral groups G1-G3 are described in greater detail in a forthcoming paper (Elfaitouri et al., accepted, Plos One).

Murine leukemia viruses (MLVs) are gammaretroviruses which may be both exogenous (transmits between individuals, i.e., horizontally) or endogenous (proviruses integrated into the germ line of mice and thereby being transmitted to the next generation, i.e., vertically). Gammaretroviruses were defined from exogenous retroviruses with MLV as a reference. The vast amount of genomic information now available shows that they are included in the large Class I ERV clade.  Figure 1 depicts the major gammaretroviral groups, based on a clustering analysis of polymerase sequences of a large number of endogenous sequences. Using MLVs (Genbank ID J02255, Locus MLMCG) as a taxonomical starting point, gammaretroviruses related to MLVs can be labeled “MLV-like retroviruses” and are here referred to as “MLLVs.” Endogenous versions of MLLVs occur in the genomes of some mammals and marsupials. The major branches segregated with previously characterized HERV groups, named after primer binding site, PBS, usage. This usage was found to be retained in the larger context (data not shown). These branches were therefore provisionally named after the respective HERV group. MLLVs are boxed in. They were defined as a cluster of polymerase (Pol) sequences which were at least 60% similar, including the MLVs. The similarity was based on the BLASTP score of their Pol amino acid sequences to each other. The concept “MLLV” is justified for the purpose of this review to encompass similar proviruses of phylogenetically distant vertebrates. Their wide distribution, in pigs, primates, rodents, and koalas, demonstrates a tendency to interspecies spread, potentially relevant both for human disease [4–9] and xenotransplantation [10–12].

The human genome also contains remnants of infections with retroviruses highly related to MLLVs, HERV-T. However, these were integrated in the rather distant past. In contrast, MLLVs have repeatedly infected nonmurine vertebrates in the not so distant past, judging from the low degree of divergence of entire proviral and LTR sequences. Mediterranean and middle Eastern cats [22], turkeys [23], gibbon apes [24, 25], and koalas [26, 27] have been “invaded” by MLLVs. This is further discussed below. In some hosts, they are both endogenous and exogenous, in others just exogenous. In the infected animals, exogenous MLLVs are associated with significant disease like encephalitis, malignancy (leukemia and lymphoma), wasting, immunosuppression, and autoimmunity. This makes it especially important to establish if also the human species is now “invaded” by a murine MLLV, that is, XMRV/HMRV. 

### 2.2. Properties

#### 2.2.1. The Gammaretrovirus Genome

**Figure 2:**
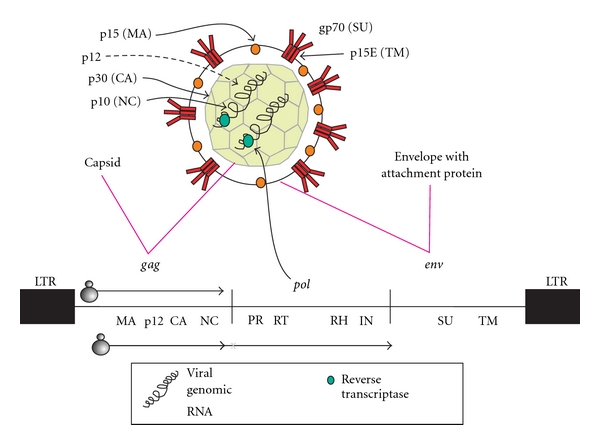
Structure and genome of a gammaretrovirus. The nucleocapsid is built from a hexameric lattice [46]. MA: matrix (p15), CA: capsid (p30), NC: nucleocapsid (p10), PR: protease, RT: reverse transcriptase (shown as a green dot), RH: RNAse H, IN: integrase, SU: surface unit (gp70), and TM: transmembrane protein (p15E). P12 is a small protein encoded from the portion between p15 and p30 in *gag*. LTR: long terminal repeat. The translation of glycoGag and normal Gag is indicated by the respective ribosome symbols. A Gag-Pol polyprotein is occasionally produced by suppression of a stop codon at the end of *gag*.

Gammaretroviruses have a simple genome (Figure 2), that is, there are no known additional overlapping reading frames for nonstructural regulatory proteins such as those which occur in betaretroviruses, deltaretroviruses, and lentiviruses. Moloney MLV is the reference gammaretrovirus [28]. Despite being simple, MLVs have some distinguishing features. The *gag* gene may have alternative translational start sites, giving rise to both a myristoylated Gag polyprotein, which contains the inner structural proteins, and a glycosylated Gag membrane protein. Many of them have a phosphoprotein following the matrix protein p15, called p12. The major Gag protein is p30, the capsid protein. It is responsible for many of the antigenic cross-reactions which gave rise to the acronym Gag (“group-specific antigen”). Further, like many other gammaretroviruses, the MLLVs have one zinc finger in the NucleoCapsid, NC, portion of Gag (the p10 protein), instead of the customary two [29]. The first finger is replaced with a highly charged sequence, binding to retroviral genomic RNA in a somewhat different way compared to two zinc finger retroviruses. The zinc finger status is here used as a taxonomic marker of a subset of gammaretroviruses [30]. All MLLVs have one zinc finger. Occasional readthrough of a Gag stop codon creates Gag-Pol polyproteins.

Another structural genomic aspect is that retroviruses with simple genomes like alpharetroviruses and gammaretroviruses occasionally may take up an oncogene in their genome, to form acutely transforming (“sarcoma”) viruses [28]. They are often replication deficient. They then need a replication competent virus, a helper virus, to replicate. The so-called murine AIDS (MAIDS) virus variants are also defective, producing a new Gag protein (p60) which contains part of the p12 protein and a T cell neoepitope [31–33]. Likewise, the feline leukemiavirus immunodeficiency-inducing defective virus (FeLV-T) has a mutated Env [34]. Immunodeficiency associated with this variant is sometimes called “Feline AIDS,” FAIDS, although the feline immunodeficiency virus can cause another form of (FAIDS). Recombination between exo- and endogenous MLLV sequences is common in both mice and cats [35]. 

MLVs can cause cancer in at least two ways, either through incorporation of an oncogene, or by integration near 5′ends of transcription units and associated CpG-rich portions [28]. The propensity to integrate into or next to promoters is a gammaretroviral specialty [36–38]. Random integration next to an oncogene is a frequent cause of leukemia in MLV-infected animals. Humans are not immune to this mechanism. MLV-based gene therapeutic vectors have the same target specificity [37, 39], see also [40]. Thus, an MLLV infecting humans would be expected to cause leukemias or lymphomas. 

Finally, the envelope proteins (Surface Unit; SU, gp70 and TransMembrane protein; TM, p15E) are central for tissue tropism, immunogenicity, and for immunosuppression. The latter contains the conserved so-called “immunosuppressive domain” (ISD) [41–45] whose mode of action is still poorly known. Thus, despite their basic structural simplicity, MLLVs can display a complex pathobiology. 

#### 2.2.2. Occurrence among Vertebrates

A rich source of vertebrate information is the collection of ERV sequences in an early version of RetroBank [47]. The program RetroTector (ReTe) [48] was used to collect more than 40.000 proviruses from whole genome analyses of thirty vertebrate genomes. ReTe is based on a pattern recognition algorithm. It uses the order of and distances between conserved retroviral motifs to detect and characterize retroviral sequences from large genomic data sets. A score is calculated from the degree of fit to a collection of conserved motifs from all seven retroviral genera. The higher the score, the better the fit to a structural model which encompasses most orthoretroviral and also some retrovirus-like sequences. A provisional genus is designated by counting the best-fitting motifs from each genus.

**Figure 3:**
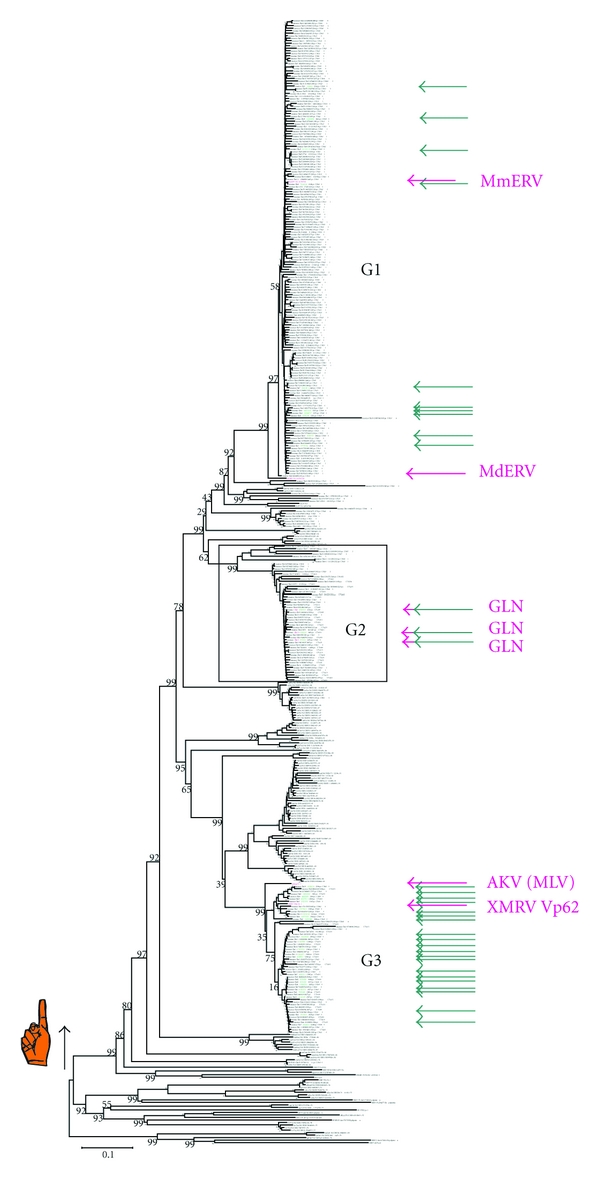
Neighbor-joining (NJ) tree based on Pol amino acid sequences of 300 high ReTe scoring MLLVs, the same as in Figure 4. The three high-scoring murine gamma groups (G1–G3) segregate in a similar way as in a *gag* nt-based tree (Figure 4). Bootstrap values are shown to the left of each branch. Structurally intact proviruses are marked with green arrows. GLN virus [15], MmERV, *Mus dunni* ERV (MdERV), AKV MLV, and XMRV (VP62 clone) are marked with magenta arrows. Murine MLLVs occur from the black arrow in the tree and upwards. The branch labels are either ERV host genus, chromosomal position, ReTe score, provisional genus, “po” for Pol, PBS, and number of stops and frame shifts (“z 0/0” means 0 stops and 0 shifts in *pol*), or a reference Pol name. PBS assignments are made by the 1.01 version of RetroTector. A more complete interpretation of PBS sequences of the G1-G3 groups is given in a forthcoming publication (Elfaitouri et al., accepted in Plos One). The major one zinc finger branch is indicated by the hand symbol. Nonmurine gammaretroviral ERV sequences were taken from the prototype of RetroBank. Further information on which assemblies were used can be found in [47].

**Figure 4:**
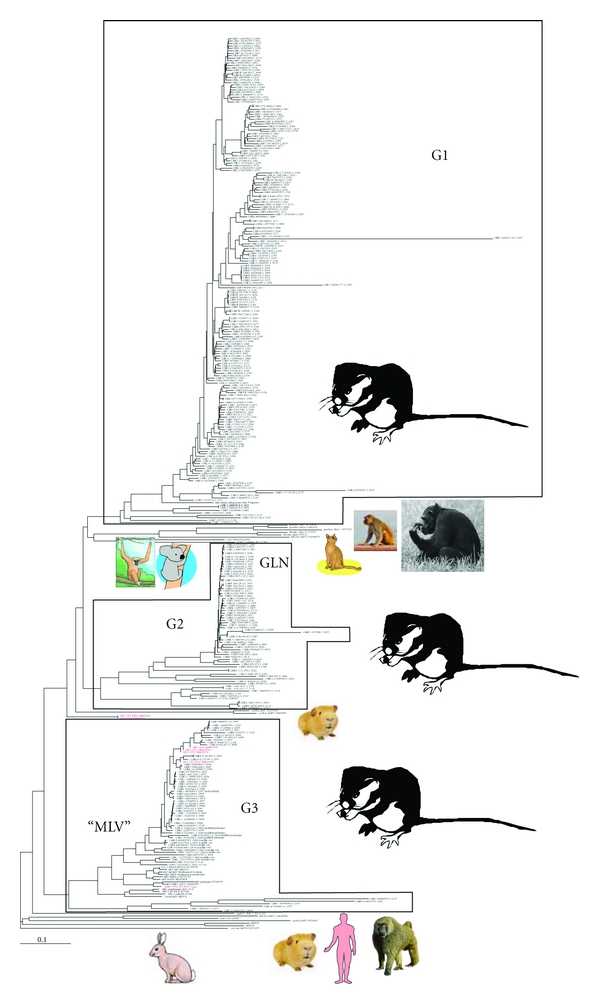
*gag* sequences of 300 high scoring mouse gammaretroviral sequences were aligned together with reference sequences. MLV sequences with ascribed tropism, from GenBank, were also added. The tree was rooted with a divergent rabbit gammaretrovirus-like sequence from scaffold 34, position 34101473 (oryCun1 assembly). Sequences in red (“HMRV”) are from the paper of Lo et al. [56]. They were from ME/CFS patients (“CFS”) or blood donors (“BD”). Two blood donor sequences from the Lo et al. study came out at the base of group G2, in other trees (not shown) at the base of group G3. The other emerged in group G3. The branch labels are either ERV host genus, chromosomal position, provisional genus (“C”: gamma), and ReTe score, or a reference Pol name. Genomic ERV sequences taken from the prototype of RetroBank were named as oryCun: rabbit, cavPor: guinea pig, felCat: cat, panTro: chimpanzee, or rheMac: rhesus macaque. Mouse sequences are just shown with their chromosomal location.

Gammaretrovirus-like sequences were detected in all of the 30 genomes (those reported in [48] plus the turkey genome). Those scoring above 1000 by RetroTector, and with only one zinc finger (*n* = 2534, from marmoset 32, dog 41, guinea pig 211, horse 4, duckbill 16, lemur 43, orangutan 82, rhesus 204, pig 79, tree shrew 11, lizard 162, cow 37, human 143, opossum 393, mouse 515, chimpanzee 192, rat 361, and zebra finch 8), were selected from RetroBank. The mouse genome assembly employed was mm8, from a C57 black mouse. Some were from the MLLV subset, as defined in Figure 1. A study of their taxonomy was initiated by clustering and consensus sequence calculation at the 85% similarity level. It resulted in 75 interhost Pol consensus sequences which together with reference Pols were used to build the tree shown in the simplified form in Figure 1. This is part of JBs ongoing work with retroviral taxonomy and will be reported in a more complete form in future papers. Especially many seemingly intact, potentially infectious MLLV proviruses were found in the mouse. In this review, we will concentrate on MLLV of mice and mention other rodents, pigs, felines, primates, and some marsupials. Of 7646 retroviral sequences detected in the mm8 assembly, 1461 were gammaretrovirus-like [47]. Some of the latter (300 proviruses) scored higher than 2000 by ReTe (Figures 3 and 4). This is a high score, achieved only by complete or virtually complete proviruses. Indeed, they all turned out to be complete proviruses with very few stop or shift (indel) mutations which could incapacitate the virus. As mentioned above, the 300 proviruses included 35 which had no such mutations. They were structurally “intact” by bioinformatic criteria. The 35 had less than 0.5% LTR divergence. They are marked with green arrows in the Pol tree presented in Figure 3. Thus, the 35 proviruses have hallmarks of being infectious and also belong to the most recently integrated murine ERVs.

Three major groups of high scoring murine gammaretroviral proviruses, named gamma 1–3 (G1–G3), were observed. 

Group G1 (188 members, 10 with open reading frame (ORF) in *gag, pro, pol,* and *env*) members encompassed the “*Mus musculus* endogenous retrovirus” (MmERV; GenBank Id AC005743 [13], as interpreted by RetroTector online [49]). *Mus dunni* ERV (AF053745) [14] is highly related. The most similar nonmouse viruses were from rat chromosomes 7 and 17 (nr4 assembly), and more distantly, gibbon ape leukemia virus (GaLV, PCGGPE) and koala retrovirus (KoRV, AF151794) sequences. 

Group G2 (59 members, 3 with ORF in *gag, pro, pol,* and *env*) contained the GLN retroviruses described by Ribet et al. [15]. It was most related to rat sequences at chromosomes 7 and 9.

A group of porcine ERVs (PERVs) located at chromosomes 9, 10, 12, and 4 (susScr10 assembly) were 74% similar to the consensus of Group G2, and 70% to the consensus of Group G1. MuRRS [18] and MuERVC [19] sequences were ancestral to groups G1 and G2 at the level of 64% similarity to their consensuses. 

Group G3 (53 members, 22 with ORF in *gag, pro, pol,* and *env*) encompassed the ampho-, eco-, xeno-, poly-, and modified polytropic MLVs [50]. Most of the MLVs which have been prominent in retrovirological research for half a century are ecotropic [51]. Amphotropic MLVs are primarily exogenous, while the others are mainly endogenous. The recombinant endogenous *Mus spretus* proviruses [16] emerged between modified polytropic and xenotropic proviruses. The HEMV provirus was at the root of the G3 branch [17].

The three major groups were discernible in trees made with several techniques resulting from alignment of nucleotides and protein sequences of the three genes *gag, pol,* and *env*. They represent three evolutionarily recent bursts of gammaretroviral proliferation in the mouse and its immediate progenitors. The third group, which includes the retroviruses reported in the human diseases, prostate cancer and ME/CFS, contains the highest proportion of structurally intact proviruses. It may thus have the greatest zoonotic potential.

In fact, the ancestors of humans were not spared infections with retroviruses related to MLLV. The so-called HERV-T is highly similar to MLLV (Figure 1). It has around 30 representatives in the human genome [52]. More distantly related are ERV-E and ERV-9W. None of the three are structurally intact in the human genome (J.B., unpublished). They are different enough from the murine G3 MLLVs to not interfere with the nucleic acid based methods for XMRV/HMRV detection (J.B., unpublished). Judging from the degree of mutational damage, HERV-T sequences integrated in a human primate progenitor genome around 30 million years ago [52]. MLLVs include the pig endogenous gammaretroviruses, PERV A, B, and C, several of which are infectious and are a problem for xenotransplantation of porcine organs to humans. The murine MLLV groups G1-3 also contain structurally intact proviruses. Very little attention has been paid to groups G1 and G2, while group G3 (“MLVs”) has been thoroughly investigated. The number of references regarding the G3 group would be staggering, and out of scope for this review. The receptors and host range of groups G1 and G2 are largely unknown. However, the GLN retroviruses seem to have a tropism similar to ecotropic MLVs [15]. Group G3 is known to contain MLLVs with several envelope-determined tropisms, see, for example, [53–55].

#### 2.2.3. Known and Probable Instances of Transspecies Transfer of Gammaretroviruses

**Figure 5:**
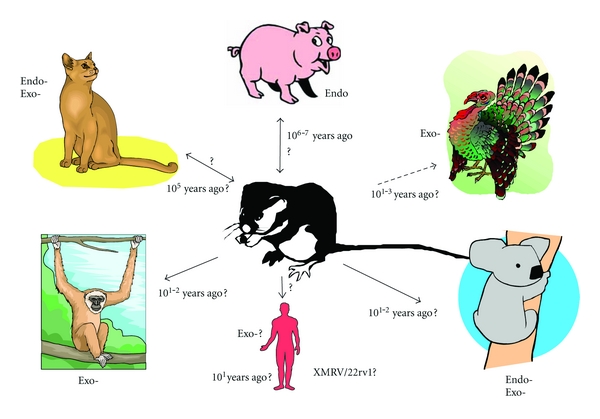
MLLVs have spread among vertebrates in recent evolutionary time. The approximate time estimates to the last common MLLV progenitor are based on references given in the text, and on the phylogenetic analysis of Figure 1. Some gibbon apes in captivity have gibbon ape leukemia virus (GaLV). Koalas have recently been infected with koala retrovirus (KoRV). More distant relatives of the murine MLVs occur in pigs and cats. Porcine MLLV ERVs (PERVs) are MLLVs but the interspecies transmission routes are uncertain. Cats have several endogenous and exogenous MLLVs, including feline leukemia virus (FeLV). Birds have recently been infected with reticuloendotheliosis virus (REV). REV is not strictly an MLLV, but is a gammaretrovirus highly related to MLLVs. Its origin is uncertain, but its closest relatives are the ERV-T-like proviruses from opossum and primates. Humans may also recently have been infected with murine MLLVs, namely XMRV, although there are now indications that this is a laboratory contamination with the 22Rv1 virus. Endo-: endogenous retrovirus. Exo-: exogenous retrovirus.

As seen in Figure 5, MLLVs can infect a wide range of hosts. For example, the XPR1 receptor, which is used by xeno- and polytropic MLVs, is common among vertebrates [57, 58]. In some cases, prey-predator relations probably contributed to the transmission [59]. In other cases, there are no such known relations. The wide range of hosts is reflected in the panorama of their receptors [55, 57, 58, 60–65]. They are known to spread via several modes: often via saliva (e.g., into wounds of fighting animals) and sexual contact [26, 62, 66–72]. Moreover, chimpanzees seem to have been infected with MLLVs from baboon and other primates [59], while baboons and cats also have common MLLVs [73]. Thus, MLLVs and similar gammaretroviruses have a tendency to spread between vertebrates. 

However, a barrier against spread to humans may be the strong anti-*α*-galactosyl antibodies in humans, which can neutralize viruses coming from species with different glycosylation patterns, like the mouse [74]. Once the virus has entered the body, its sugars will follow the human glycosylation pattern, and the virus will not any longer be neutralized. Therefore, this barrier is not absolute. 

A variety of other restrictions, like the APOBEC cytidine deaminases [50, 75, 76], tetherin [53], and TRIMs [77–79] also affect retroviral spread between species. However, restrictions may be as important within a natural host as between hosts [80, 81]. The high XMRV replication in the cell line 22Rv1 [82] and the ready growth of XMRV in LNCap cells [83], both RNAse L-deficient human prostate cancer cell lines, plus the ability to grow in human PBMCs [84], indicate the ability of XMRV to grow in human cells [84] and the importance of an intact interferon system for the defence against it. These barriers to spread could probably be overcome, and humans be infected by XMRV, although the infectivity *in vivo* is hard to predict. It was recently reported that XMRV can grow in human PBMCs [84].

Judging from the wide spread of MLLVs, a zoonotic spread of XMRV/HMRV from mouse to human, directly or indirectly via another vertebrate, is not impossible. Humans are occasionally exposed to animals which harbor MLLVs. For example, microbes known to spread to humans from pets are viruses (arena-, hanta-, pox-, orthomyxo-, and rhabdoviruses), bacteria (chlamydiae, salmonella, tularemia, and leptospira), protozoa (toxoplasmosis), and helminths (worms). Rabbits, mice, rats, and guinea pigs are frequent as pets. The frequency of animal contact should therefore be recorded in epidemiological investigations regarding MLLVs, like XMRV/HMRV, in humans. 

Like other MLLVs, a human MLLV would be expected to spread via kissing, sex, intravenous drug use, blood donation, and possibly via breast feeding. Enough systematic tests for MLLVs in the corresponding body fluids have not been performed. There should also be an overrepresentation of XMRV/HMRV in intravenous drug users and in patients infected with other sexually transmitted microbes, like HIV [85, 86]. This needs more study. Credible transmission chains between ME/CFS patients (with the exception of outbreaks), between PC patients, from ME/CFS to PC, and from PC to ME/CFS have not been reported (cf.  Table 1).

## 3. Did MLLV Spread Zoonotically to Humans?

Gammaretroviruses related to the MLV were found 2006 in a few percent of patients suffering from prostate cancer [9]. They were initially named XMRV. In 2009, XMRV was also found in patients suffering from ME/CFS [6]. In 2010, the term XMRV was replaced with HMRV, because gammaretroviral sequences found in ME/CFS were found to be more diverse than just XMRV [56].

### 3.1. The Findings in PC

In 2006, Urisman et al. reported the discovery of a novel retrovirus in a subpopulation of prostate cancer patients in the United States [1, 8, 9]. Using fluorescent in situ hybridization, the viral nucleic acid was located to stroma cells, not the cancer cells. However, others found it also in the cancer cells [110]. This retrovirus was identified by means of a DNA microarray (“Virochip” [8, 9, 111]) screening of known cancer samples. The DNA microarray contained 11 000 pieces of 70 bp long oligonucleotides from approximately 950 evolutionarily conserved viral genome sequences. It has been used to screen for the presence of viral DNA and also identify which family the detected virus belongs to. Its success is a practical demonstration of the utility of a phylogenetically directed search for new viruses. The prostate cancer results are covered by other reviews in this volume. Only selected data will be discussed here.

#### 3.1.1. If XMRV/HMRV Is Not Situated in Cancer Cells, How Could it Contribute to PC?

MLLVs are not associated with prostate cancer in animals. Therefore, the existence of an MLLV in human prostate cancer is unexpected. Initial reports indicated that the retrovirus was integrated into stromal cell fibroblasts in the prostate cancer samples. This could present a problem for the hypothesis that the newly discovered virus is part of the oncogenic process. However, it is generally accepted that there are close interactions between the epithelial cells and the underlying stromal cells, such as fibroblasts, smooth muscle cells, endothelial cells in blood vessels, and pericytes, both in normal tissue and in tumours [112, 113]. This interaction consists of both paracrine signalling and interactions with the extracellular matrix, which *in vivo* have been shown to have a significant impact on the growth of transformed cells. It was shown 1966 that polyoma-infected epithelial and stromal cells would not transform unless grown in contact with the underlying stroma [114, 115]. It has also been shown in both *in vivo* and *in vitro* studies that tumour-associated fibroblasts contribute to the transformation of immortalized epithelial cells [116], indicating that there are permanent changes in the stromal cells in tumours. What is even more interesting is that it has been shown that the tumour-associated fibroblasts can cause nontumourigenic prostate epithelial cell lines to transform if cocultured [117]. Thus, one cannot dismiss an MLLV as being uninteresting with regard to tumourigenesis in the prostate.

### 3.2. The Findings in ME/CFS

The main symptom of CFS is a persistent and debilitating fatigue with rapid onset. It often commences after an episode of influenza-like symptoms. The afflicted patients were previously healthy [87]. The symptoms include fatigue, loss of memory or concentration, sore throat, painful lymphadenopathy, muscle pain, headache, unrefreshing sleep, and extreme exhaustion after exercise. The cause of CFS is not yet known. It is in need of clinical and laboratory studies for its further definition. ME/CFS can, however, be diagnosed according to internationally accepted clinical criteria, see, for example, [118]. It seems to be a rather common disease, maybe amounting to 0.5% of the population [118]. Finding the cause, new diagnostic techniques, and, hopefully, a cure, for this often debilitating disease is a high medical priority. ME/CFS borders to the diseases fibromyalgia (FM) and irritable bowel syndrome (IBS). FM-like chronic pain and IBS-like intestinal symptoms are common in ME/CFS [119]. 

In 2009, Lombardi et al., in Judy Mikovits' group, reported the discovery of the XMRV virus, using PCR and serology, in 67% of chronic fatigue syndrome (ME/CFS) patients compared to 3.7% in healthy controls [6]. Reports which verify [56, 102, 103] and do not verify [84, 88–93, 105, 106, 108, 120] the original ME/CFS report have come. The situation is volatile and cannot be extensively covered here. The conflicting results may be due to methodological differences, an uneven geographic distribution of XMRV/HMRVs, or viral genetic variation. Switzer et al. used a Western blot with a lysate from MLV, another group G3 virus, to examine antibody response in 51 CFS patients and 53 healthy donors. No specific reaction was seen [105].

In a UK study involving 170 CFS patients as well as 395 non-CFS patient controls, 4.6% of the samples contained neutralizing antibodies, but only one of these was from a CFS patient. Most of these positive sera were able to neutralize MLV virus particles pseudotyped with Env-proteins of other viruses indicating significant cross reactivity [90]. Hohn et al. searched for antibody activity of 36 CFS and 112 MS patients and 27 healthy controls using an Env-ELISA and a capture Gag-ELISA. No evidence of specific seroreactivity was found [84].

#### 3.2.1. Could an MLLV Cause the Combination of Neuro- and Immunopathology Which Occurs in ME/CFS?

MLLV infections are sometimes associated with immunosuppression [121]. ME/CFS patients have a degree of immunosuppression, neurological symptoms, and often an enteritis. Giardia has been associated with ME/CFS [122, 123]. Mice with MAIDS are especially sensitive to intestinal parasites like Giardia, a similarity which may be fortuitous [122, 123]. Likewise, cats with FAIDS are also immunosuppressed and develop enteritis. Although such correlations are diffuse and may be spurious they indicate that infectious agents could give ME/CFS-like disease.

### 3.3. Other Diseases Expected to Occur If There Is an Infectious Human Gammaretrovirus

Murine and feline diseases where MLLVs are known to play a major role are leukemia, lymphoma and encephalitis (and other neurological diseases) and, as mentioned, immunodeficiency [124–132]. Autoimmune disease has also been linked to MLLVs [133, 134]. MLLVs should thus be searched for in these diseases. So far, studies of associations of XMRV/HMRV with such human diseases have not been published.

### 3.4. Can Phylogeny Direct Our Efforts to Detect MLLV Infection in Humans?

#### 3.4.1. Viral Nucleic Acid Detection

Nested PCRs have been used in several of the positive XMRV reports. Such PCRs have a high sensitivity, but also a high risk of amplicon contamination. 

The lower sensitivity of microarray analysis makes it less susceptible to contamination. This makes the initial serendipitous observation of XMRV in prostate tissue [9] especially credible. Although PCRs are more sensitive, they may miss imperfectly matching targets. High sensitivity also means high risk of contamination. Amplimers, synthetic sequences, and plasmids containing target sequences are notorious problems. However, also genomic DNA harboring target sequences (ERVs) is a problem. Mouse DNA is a special case, because it contains many HMRV PCR target sequences per copy of mouse genome (see above). Thus, each XMRV/HMRV PCR should be evaluated for its detection range and tendency to give false positive results due to mouse DNA contamination. Several of the PCRs which have been used to detect XMRV react also with mouse DNA. Some, like the integrase-based PCR of Ila Singh [135], do not react with mouse DNA unless the DNA is present in high concentration. Others react strongly with mouse DNA. Mouse DNA contains at least two sequences which are highly similar to XMRV, with ORF in gag, pro, pol and env: Proviruses at chromosome 1, position 172778230 and chromosome 5, position 23221036. The similarity is obvious in *pol* and *env*, less so in *gag* (mouse genome assembly mm8, which comes from C57Black). These proviruses are situated next to XMRV VP62 in the Pol-based tree in Figure 3. All broadly targeted (“HMRV-specific”) PCRs react with mouse DNA, while some “XMRV specific,” like the Singh PCR, do not react, or react only with high amounts of mouse DNA (Elfaitouri et al., submitted). Among 30 vertebrate genomic DNAs analyzed bioinformatically, mouse DNA is most likely to give such spurious PCR signals (Blomberg and Elfaitouri, unpublished). 

A test which lends high credibility to a positive provirus detection is if integration sites in the host genome can be cloned and sequenced. This has been reported for XMRV in PC [7]. However, Garson et al. [99] claimed that integration sites in 2 of 14 prostate cancer patient samples reported from Silverman's group [8, 9] were identical to those of the experimentally infected human tumour cell line DU145 used in the same laboratory. This raises the possibility that this finding is due to contamination.

**The Problem of Mouse DNA and XMRV DNA Contamination. Where Does It Come from?**

Several laboratories have reported frequent occurrence of mouse DNA in samples from humans. Some of the PCRs used for detection of XMRV/HMRV also become positive when mouse DNA is present, because (as explained earlier in this review) the mouse genome contains many proviruses which react in these PCRs. However, some XMRV PCRs do not react with mouse DNA [110]. They should be less likely to give false positive signals due to presence of mouse DNA. Presence of mouse DNA in human samples may sound absurd and is often not easy to explain. It may be caused by contamination of chemicals and biologicals used to prepare the samples. For example, mouse DNA may be present on microtomes used for preparation of tissue sections from both mouse and humans. Further, some cell lines (especially hybridomas of mouse origin) contain high amounts of XMRV or related MLVs and can contaminate other cell lines [136, 137]. It is therefore logical that murine monoclonal antibodies, including anti-Taq polymerase antibodies, which are used to provide a “hot” start in PCRs, sometimes contain traces of nucleic acid from MLVs [101]. Moreover, the highly XMRV producing cell line 22Rv1 contains a virus which is very similar (essentially identical; see Figure 4) to reported XMRVs [99]. It is a human prostate cancer cell line which was infected with XMRV during passage in nude mice [2]. According to recent information, XMRV arose by a unique recombination event between two defective MLV sequences [2]. It is therefore reasonable to assume that XMRV originated from the 22Rv1 virus which then has been a contamination source of many of the published positive results from prostate cancer and ME/CFS. In fact, all cell lines which have been passed in nude or SCID mice should be suspected of retroviral contamination.

If contamination explains all positive PCR results in ME/CFS and prostate cancer, why would the frequency in ME/CFS patients be at least tenfold higher than in the controls? The patient samples may have been more frequently opened than the control samples. At least two kinds of contamination, with mouse and XMRV DNA, respectively, have to be invoked. Although false positive PCR results due to contamination in the laboratory is a frequent event, contamination on such a grand scale is beyond previous experience.

There are similarities between the XMRV/HMRV and the “Human Retrovirus 5” (HRV5) stories [138]. HRV5 is one of the so-called “rumor viruses” [139]. It turned out to be a defective rabbit betaretrovirus (RERV-H) whose DNA is abundant in rabbit sera [140]. The rabbit genome contains around 700 copies of RERV-H [140]. Any laboratory which handles rabbit sera is at risk of RERV-H contamination. In analogy with this, a low level of mouse (or XMRV) DNA could be present in laboratory reagents, cell cultures, or in the laboratory environment, as evidenced by many confirmed reports of contamination, or unconfirmed reports of human retroviruses [73, 93–96, 99–101, 136, 137, 141–165]. Thus, extensive contamination controls must be performed if PCR is used for detection of XMRV/HMRV.

#### 3.4.2. Viral Reverse Transcriptase Detection

Reverse transcriptase (RT) activity is a fundamental and conserved function of retroviruses. Detection of RT activity is an established method for retrovirus discovery and diagnosis. There is an evolutionary limit to the extent to which the enzyme can mutate. Presence of significant retroviral RT activity in a biological sample is thus not only an indication of virus protein expression, but of retrovirus replication. RT activity assays thus give an additional dimension compared to detection of viral nucleic acids or proteins. The RT enzymes from different retroviruses have different enzymatic properties. By varying pH and the composition and concentrations of certain components, it is possible to optimize assays for a virus family or subgroup [166]. RT assays can also be performed with PCR readout, which gives a very high sensitivity [167, 168]. Quantification of RT activity may be complicated by a myriad of RT inhibitory factors and requires enzyme purification. A simple colorimetric RT activity assay [166] can be used both for monitoring propagation of XMRV virus in cell culture and for direct detection in samples from humans [169]. RT assays are somewhat less sensitive than PCR and have less problems with contamination and a broader detection range. An RT assay optimized for XMRV has been developed (http://cavidi.se/). It has a minimum level of detection of 0.01 *μ*U RT activity, which corresponds to approximately 50 virus particles per reaction (Elfaitouri et al, accepted for publication in Plos One). It is currently used as an independent technique for following isolation and propagation of MLV-related viruses in our laboratory.

#### 3.4.3. Virus Isolation

The Mikovits group at the Whittemore Peterson Institute reported a high frequency of virus isolation from ME/CFS patients [6, 102, 103]. We are not aware of a report on XMRV isolation from prostate cancer patients. Virus isolation is inherently less susceptible to contamination than PCR is, because retroviruses are easy to disinfect. They also lose infectivity after drying. The virus isolation results are therefore the mainstay of the proponents of XMRV [102, 103]. An especially efficient and specific virus isolation test seems to be the DERSE (detector of exogenous retroviral sequences) assay [170]. The isolation results from ME/CFS patients were recently contested [3]. Patients which earlier were reported to be XMRV isolation positive were found to be negative on retesting. This raises the possibility that also the virus isolation results were due to contamination with 22Rv1 tissue culture virus. The 22Rv1 virus is present in billions of copies per mL of tissue culture supernatant. Retroviral contamination of cell cultures is common, see, for example, [136, 137, 171]. Low or moderate expression of potentially infectious ERVs in cell culture is a particularly vexing problem [137, 143, 172–176].

#### 3.4.4. Serology

Antigens for use in XMRV/HMRV serology range from synthetic peptides to recombinant proteins. In this situation, cross-reactive epitopes in Gag and transmembrane proteins are of special interest. Epitopes from, primarily, Env and Gag proteins of XMRV and related MLVs should be covered. It is fortunate that much information regarding B-cell epitopes is available for MLVs and FeLVs (against which an effective vaccine exists). It can guide the selection of synthetic peptides. Synthetic peptides, primarily mimicking linear epitopes, have been useful for development of serological assays for detection of other retroviruses such as HIV and HTLV [177–182]. However, an optimal serological assay should cover both linear and conformational epitopes. Whole virus [105, 109, 183, 184], recombinant XMRV proteins [84, 104, 106, 109, 183, 185], and a “surrogate” spleen focus forming virus (SFFV) fluorescence-activated cell sorter (FACS) antibody test [6] have been used. SFFV has an envelope which is a recombinant between the envelopes of the infectious Friend helper virus and an endogenous polytropic virus. It also has a large deletion involving the SU/TM cleavage site. Cells with and without transduced SFFV envelope are incubated with serum and fluorescent antihuman IgG and then run in an FACS. This is an elegant and specific technique, but is dependent on a few cross-reactive envelope epitopes. Western blot (WB) using whole virus (or a set of recombinant proteins) [105] is a *de facto* serological golden standard in clinical retrovirology. It would be desirable to have a confirmatory WB assay for serological XMRV diagnosis. Screening assays could be existing enzyme immunoassays (EIAs), suspension arrays [120], or chemiluminescent magnetic microparticle immunoassays (CMIA) [109]. Neutralization assays [89] can be highly specific, but may be too narrow if a broad search is desired. The FACS analyses of antibodies binding to SFFV envelope transduced cells should also be very specific. None of the latter two are, however, suitable for large-scale screening. There is a long history of serological findings of gammaretrovirus antibodies and antigens in human disease [73, 186–203]. Given the tendency of serological methods to cross-react, weak serological reactions to a few epitopes alone are not strong evidence. 

A serological diagnosis of a retroviral infection needs demonstration of an immune response at several epitopes. It cannot be the only serological evidence. Ideally, several tests with different specificities, covering both broadly cross-reactive and specific, linear and conformational, epitopes should be used. Several of these serological assays are capable of detecting antibody reactivity to other MLVs. Thus, although it may not be intentional, the intense hunt for XMRV antibodies is also a hunt for other MLLVs in humans. The largely negative outcome [84, 105, 106, 108, 204] may be taken as evidence against widespread MLLV infection in humans.

#### 3.4.5. Why Was XMRV/HMRV Not Discovered in the Virus Cancer Program during 1964–1977?

A considerable US effort to find new retroviruses was the Virus Cancer program [205]. Virus isolation and reverse transcriptase assays were main techniques. MLV and similar viruses are relatively easy to cultivate. They were known at the time and were also main targets for the program. It is therefore notable that such a virus was not detected in humans with cancer during this project. Reasons could be manifold, either due to bad luck, inappropriate methods, due to an introduction of XMRV/HMRV into humans, or spread of a tissue culture contaminating virus after the conclusion of the Virus Cancer program.

#### 3.4.6. Summary of Current Controversies Regarding XMRV/HMRV

Since the initial reports of XMRV in US patients with prostate cancer and CFS, several research groups have been attempting to replicate these results. Especially, the connection of XMRV to CSF has raised considerable interest in this virus. However, it has not been possible to detect the virus in studies from China where Hong et al. have analyzed samples from 65 CFS patients and 85 healthy controls [92], from Netherlands where van Kuppeveld et al. investigated samples from 76 CFS patients and 69 matched controls [98], from UK where Groom et al. tested samples from 170 CFS patients and 395 non-CFS control patients [90], and Erlwein et al. investigated samples from 186 CFS patients [89], and from the US where Switzer et al. analyzed samples from 51 CFS patients and 53 healthy controls [105], using either PCR-based methods for detecting viral RNA/DNA or methods for detecting neutralizing antibodies against XMRV. Hohn et al. were likewise unable to detect XMRV in any of the 589 German prostate cancer samples they analyzed for the presence of XMRV [84, 106]. To further confuse the issue, a US research group failed to detect the XMRV itself but was able to detect viral sequences closer related to other MERVs (“HMRV”; here shown to belong to group G3 MLLV) in a retrospective study of blood samples from CSF patients [56].

#### 3.4.7. An Attempt to Reconcile Current Results Regarding XMRV/HMRV with Each Other

**Why Is XMRV/HMRV So Hard to Detect by PCR and Serology?**

A possibility which could reconciliate the conflicting findings could be that a chronic XMRV/HMRV infection becomes progressively harder to detect both by nucleic acid, virus isolation, and serological methods. XMRV/HMRV would then establish a low-grade infection in a limited number of cell types, with a waning immune response. This is reminiscent of what was seen in experimentally XMRV-infected macaques [109, 183]. The dynamics of antibody response elicited by XMRV were studied in five XMRV-infected macaques. Using recombinant gp70, p15E, and p30 in western blots and CMIA, Qiu et al. found evidence of antibodies two weeks after infection. The antibodies persisted for at least 158 days. Although all three proteins elicited an immune response, antibodies to recombinant gp70 and p15E showed higher sensitivity than p30 [109, 183]. The Western blots were very clear. Such XMRV Western blots were never reported from humans. There was a tendency for the antibody levels to decrease over time. Stimulation with a dose of XMRV antigen gave rise to a burst of viral replication and a rise in XMRV antibodies. In another study, *Mus pahari* was experimentally infected with XMRV [184]. Antibodies to XMRV Env (p15E and gp70) and Gag (p30) were detected in Western blots and in ELISA tests. Neutralizing antibodies also developed.

Thus, the expected course of an XMRV/HMRV infection is an initial phase with intense viral replication, easily detected by PCRs on nucleic acids from plasma or PBMCs, followed by development of a strong antibody response which can be demonstrated by several kinds of serology, and a full WB pattern, similar to the situation during HIV infection.

However, the difficulties of finding both viral nucleic acid in blood samples and a weak or nonexistent antibody response in virus isolation or XMRV/HMRV PCR-positive persons lead us to two alternate interpretations.

First, we must hypothetically consider a model of low-grade chronic XMRV/HMRV infection of humans. Such a “stealth” infection could have similarities with ERVs. The immune response to ERVs is abnormal, possibly because they are perceived as “self” by the adaptive immune system [206]. Their protein expression may also be weak. In putative “stealth” non-ERV RV infection a low degree of continuous antigenic stimulation could lead to a low, and waning immune response [207]. Although a vigorous antibody response is the most common reaction to a retrovirus infection, a very weak antibody response is also seen in some cases of HLTV-2 infection, which can also be accompanied by a low degree of viral replication [208, 209]. It is a considerable difficulty for the diagnosis of HTLV-2 infections in humans by PCR and serology. Likewise, HIV patients which were treated early during the infection may develop an abortive immune response [210, 211]. It is conceivable that the XMRV/HMRV situation could be similar. There are aspects of the MAIDS/FAIDS models which fit with ME/CFS and this model.

The second alternative is that all reports of XMRV/HMRV in humans have been due to contamination or serological cross-reaction. The PCRs could have been confounded by various forms of contamination (see below). The positive serologies in ME/CFS patients have largely been from the surrogate SFFV FACS antibody test [6], which alone does not fully suffice as evidence. It would be a sad outcome of a fascinating and important story.

### 3.5. Medical and Ethical Consequences of the Uncertain Diagnostic Situation

The finding of XMRV/HMRV in ME/CFS has far-reaching implications, for the personal life of the patients (sex, kissing, breast feeding, etc.), for the development of diagnostic methods, for transfusion safety, and for the understanding of other human diseases with a possible retroviral etiology. It is reasonable to demand that measures to protect blood transfusion recipients from infection are as rigorous as the sensitivity and specificity of available tests allow. In the situation of today, where the reported frequency of XMRV infection found with different methods in blood donors or comparable healthy individuals varies from 0 to 7%, there is no simple test strategy available. However, the mere suspicion that a retrovirus like XMRV is frequent in patients suffering from ME/CFS is a basis for abstaining from using such patients as blood donors [212]. The finding of XMRV in PC also raises medical and ethical questions. However, the frequency of positivity is at a few percent, and claims of a connection have not reached the high frequencies reported in ME/CFS (>60%). 

Both ME/CFS and PC patients suffer from the uncertainty regarding XMRV/HMRV positivity in the two diseases. The patients must make personal decisions regarding sexual contacts, kissing, and breast milk feeding of their children. Few PC patients will know their alleged XMRV/HMRV status, but the rather widespread testing of ME/CFS patients for this virus has created a group of patients who are left in limbo. The temptation to start antiretroviral treatment [213] despite the current scientific controversy can be hard to resist.

## 4. Conclusions

Research on XMRV/HMRV in humans is evolving rapidly. There is a great need for confirmation of the reports on XMRV/HMRVs in PC and ME/CFS. In view of the recently demonstrated diversity of retroviral sequences in ME/CFS, it is also important to establish the detection range of XMRV/HMRV detection methods. Contamination of cell cultures with 22Rv1 virus and PCRs with MLV nucleic acid and mouse DNA is known to occur. Whether all reports on MLLVs in humans can be explained by them is uncertain, but not unlikely. The XMRV/HMRV story has both credible and less credible aspects (Table 1). The original XMRV detection in prostate cancer was serendipitous and made with several independent techniques, together forming a credible case. The proven integration into human DNA was especially convincing. The finding of XMRV/HMRV in ME/CFS also has a credible aspect; the immunomodulating properties of MLLVs could theoretically explain the disease. However, the epidemiology of XMRV/HMRV transmission still is unclear. The absence of an easily measurable immune response is also a memento.

We conclude that MLLVs are widespread as ERVs among vertebrates. There are many signs of interspecies transmission of MLLVs. However, only a few of the MLLVs are structurally intact. The mouse genome is unique in its high content of MLLVs. It contains three major MLLV groups, of which two (G1 and G2) have not hitherto been reported. Group G3 contains the MLVs and all or nearly all of the MLV-like retroviruses which have so far been detected in humans, that is, XMRV and HMRV. 

The study of XMRV/HMRV is important from a range of perspectives, one of which is screening of blood donors for potentially harmful pathogens. Xenotropic viruses also raise concerns regarding research into xenotransplantation of organs [16]. 

The detection of human infection with XMRV/HMRV has proven to be difficult. This may either be due to absence of the virus or to a low-grade infection, with a minimal viral replication and a minimal serological response. Although that goes contrary to expectations, such a situation sometimes occurs in HTLV and HIV infections.

## Figures and Tables

**Table 1:** How well does XMRV/HMRV fulfill the expected properties of an MLV spread to humans + means an argument for, and—an argument against expectation?

Property	Expected finding	Observed	Conclusion, for or against XMRV/HMRV in humans?
		+Hybridization array w. conserved viral 70-mers, an insensitive technique [9]. +Serendipitous.	For?
	Viral nucleic acid	+PCR (positive, XMRV [87], positive, HMRV [56] and −negative [3, 85, 88–100] results). −Proven or suspected contamination w 22Rv1-like virus and mouse DNA [3, 57, 93–96, 99–101].	Against?
		+Integration into host genome [8]. −Some of the integration sites occur in an XMRV-infected cell line [99].	Against?
Discovery/followup		+Cloning of XMRV into infectious clone (PC, ME/CFS) [8]. −High sequence similarity and unique recombination suggest contamination with DNA from XMRV producing cell line [2].	Against?
	Virus isolation	+Virus isolation (ME/CFS patients) [6, 102, 103]. −High sequence similarity, lack of evolution, and unique recombination suggests contamination from XMRV producing cell line [2, 82]. −Isolation results cannot be reproduced [3].	Against?
	Antiviral immune response	+SFFV FACS (ME/CFS) concordance with PCR outcome [6, 102], +ELISA (PC, ME/CFS), positive [104] and −negative [84, 92, 105–107] outcomes, −Virus neutralization, negative outcome [90], −Western blot (ME/CFS) negative outcome [105, 108], −CMIA (ME/CFS), negative outcome [109], −T-cell response, negative outcome [86].	Against?

Epidemiology, early phase	Contact with mouse	Not observed/reported	Against?

Epidemiology, late phase	Human-human spread, via saliva, sex, and mother-child	−Although occasional ME/CFS outbreaks occur, most cases are sporadic, with no spread between spouses. −No reported epidemiological link between prostate cancer and ME/CFS −No known overrepresentation in STD and in IVDU [85].	Against?

Pathogenesis	Leukemia, lymphoma	Prostate cancer?	For or against?
	Immunodeficiency	Immunodeficiency?	
	Encephalitis (MAIDS)	Myalgic encephalomyelitis?	
	Enteritis (MAIDS, FAIDS)	Irritable bowel syndrome?	
	Autoimmunity	Thyroiditis?	

Replication	Relatively easy to detect in blood	Hard to detect in blood.	Against?

Immune response	Strong B-cell response, positive WB	Weak or absent B-cell response.	Against?
